# Planning to fail? How science can respond to reduced climate mitigation ambition

**DOI:** 10.1038/s44333-024-00002-8

**Published:** 2024-05-22

**Authors:** Greg Marsden, Tim Schwanen

**Affiliations:** 1 Institute for Transport Studies, University of Leeds, LS2 9JT Leeds, UK; 2 Transport Studies Unit, School of Geography and the Environment, University of Oxford, Oxford, UK

**Keywords:** Energy policy, Geography, Climate-change mitigation

## Abstract

The prospect of remaining within 1.5C of planetary warming relies on developed economies tracking increasingly steep and challenging emission reduction pathways. This paper explores how the UK is now proactively planning to miss its targets, using the surface transport sector as a critical case. It discusses how the research–policy interface might both challenge downgraded ambition and provide more actionable routes forward.

The UK Government has staked a claim as a global leader in climate policy, being the first national government worldwide to establish a Climate Change Act (2008), which mandated an 80% reduction in climate emissions by 2050. It updated this act in 2019 to 100% net emission reductions, the so-called ‘Net Zero’. Progress has been made, in particular in decarbonising the power sector, which has underpinned a meeting of carbon budgets in the period up to 2023^1^. However, as policy change now requires actions across all sectors at pace, the Government is currently off track to meet the budget goals out to 2037. This is particularly problematic for the transport sector as surface transport (excluding domestic and international maritime and aviation) saw no reductions in CO_2eq_ emissions from 1990 to 2019. It is the largest contributor to total emissions at 26% (109MtC), despite reductions in car use since the COVID-19 pandemic^2^.

In 2021, the UK Government published its first Transport Decarbonisation Plan (TDP), which suggested multiple policy pathways and considered the possibility of going further and faster than the Government’s Advisory Body, the Climate Change Committee (CCC), had recommended a year earlier^3^. Yet, the 2021 ambitions have been watered down. By March 2023, the TDP pathways had been superseded by a pathway that falls well behind that proposed by the CCC in 2020, and most of the policies to reduce emissions by influencing travel demand had been removed^4^. In September 2023, Prime Minister Rishi Sunak announced the intention to push back the dates for the phase-out of the sale of new fossil fuel-powered vehicles from 2030 to 2035. He also attacked devolved and local government initiatives on lower speed limits, car sharing, 15-min cities, and traffic calming for residential areas. A “Plan for Drivers” accompanied this policy shift, declaring that the UK “can decarbonise and maintain our freedoms”^5^.

In this short commentary, we reflect on why a simultaneous shift in problem framing, politics, and policies has occurred. UK transport policy offers, we suggest, an early example of how strategies that are developed to meet the increasingly challenging carbon budget obligations may fare when faced with the politics of implementation. We conclude by exploring the role research plays in influencing change in one of the defining challenges of our lifetimes.

## Policy change and Kingdon’s three streams model

Our paper takes inspiration from political theorist John Kingdon’s three streams model of policy change^6^. In it, he argues that there are three separate but inter-related streams that need to be aligned during so-called ‘policy windows.’ The first stream is ‘problems’, which relate to the recognition of an issue as important relative to others through reports, indicators, or failures that show that an issue needs attention. In our case, the climate challenge is well documented and formally recognised in legislation. The second stream is ‘solutions’, where a set of recognised and acceptable policy options needs to be available to address the problem in ways that make its formal recognition politically salient. The third stream is ‘politics,’ where there needs to be sufficient alignment with the politics of the day and, relative to tackling other issues across government, the political benefit from making a change must outweigh the perceived challenges and risks of adopting a new solution or policy. Our discussion revolves around a case where previously ‘necessary’ solutions are removed from the policy arena rather than how new policies come about. We, therefore, pay less attention to other aspects of the three streams framework, such as ‘policy entrepreneurs’, who work to get things on the agenda and draw together the streams to deliver change. We refer readers to reviews discussing Kingdon’s approach and the breadth of its application^7^, as well as its utility for transport and climate policy analysis^8^ for further detail.

## Problem recognition: the equivalent of 10 pandemics

The recent shifts in UK transport policy have significantly increased an already monumental CO_2eq_ emissions reduction challenge. A pathway for transport consistent with the country’s legal decarbonisation targets requires some mix of measures that reduce how much people travel by car, increase the use of alternative modes of transport, and shift away propulsion from fossil fuels completely^1,9^. Part of this mix is a mandated ending to the sale of new fossil fuel-powered vehicles by 2035 and for 80% of new car sales to be zero emission by 2030^10^. This locks in key aspects of the emissions reduction pathway, which, while ambitious, still lags behind what the CCC anticipated in 2020. As travel demand reduction and mode shift have been removed almost entirely from the anticipated policy pathway, the CCC now estimates that annual emissions reduction gaps of 22.7 MtC for 2023–2028 and 19.2 MtC for 2028–2032 have arisen^1^. Marsden^11^ estimates that the downgrading of policy ambition has resulted in a 224 MtC overshoot compared to the CCC’s plans from 2020. Since the technology pathway has now been legislated, this overshoot would have to be compensated through travel demand and mode shift actions, notwithstanding their downgrading in recent national policy. To put the emissions gap in context, the reduction in emissions between 2019 and 2020, when large parts of the UK economy were closed down due to the COVID-19 pandemic, was 24 MtC^1^. In other words, the scale of required travel behaviour change equals the impacts of around 10 consecutive years of pandemic-scale traffic reductions. The mechanisms for formal problem recognition now serve to ratchet up the pressure to act.

## Politics, reframing the problem and recasting the solutions

In the context of an increase in the recognised scale of the problem, the recent change in Prime Minister Sunak’s messaging in support of motorists is clearly a political choice to push back the need to act and to reject a substantive travel reduction strategy. The legitimisation of that choice, Kingdon’s three streams model suggests, requires reframing the problem, attacking the previous solutions (which sought to solve the ‘wrong problem’), and proposing some alternative actions. All three strategies are evident.

Consider problem framing. Acting on Net Zero has overwhelming public support, with recent polling suggesting that 70% of the public support the goal of being Net Zero by 2050, against 17% who do not^12^. Sunak and his team therefore decided to reframe the decarbonisation problem as less urgent and earlier action as being socially regressive. The subtle reframing of the UK’s climate commitments as being about 2050 and not about the total carbon budget left to consume is at odds with climate science^13^ but plays to decision-makers’ and the public’s lack of detailed understanding of it.

Kingdon’s work suggests that policies are only recognised and enacted when they are deemed capable of tackling the problem in ways with which politics can cope. This, we believe to constitute the biggest challenge for transport and one which may well spread to other policy domains and geographical contexts. Because the scale of change required now equals ‘10 pandemics’ and meaningful demand reduction is marginalised further, the policy mix to stay on transport sector pathways that are commensurate with the UK’s legal obligations becomes more difficult to imagine. The reframing of solutions that accompany the problem reframing is one that enables slower progress on electrification and explicitly repositions demand reduction policies as unnecessary, undemocratic, undesirable, and/or unfair. All of these strategies have been deployed in more recent policy announcements.

Of course, decarbonisation policy is developed and implemented in a multilevel governance setting and not wholly decided by the UK Government in Westminster. There are devolved national governments in Scotland, Wales and Northern Ireland, and local authorities also play critical roles, particularly regarding implementation and in major cities^14^. The Scottish Government has set a target for a 20% reduction in car kilometres by 2030 and the Welsh Government a reduction of 10% per capita. Local authorities declared climate emergencies and had goals for zero emissions from transport as early as 2028. Policy ambitions and political commitment to transport decarbonisation remain at these levels, at least for now. However, actions and achievements on the ground are already lagging behind ambition^1^, and meeting local targets will only become more difficult in the absence of a whole-UK approach because of the importance of national taxation regimes to transport decarbonisation and the heavy dependence on devolved administrations and local governments on funding allocations from Westminster. Worse, lower-tier governments now need to counter the narrative that their climate policies are part of a 'war on motorists' and seek to penalise 'hard-working families'.

## Lessons

The experience of recent UK transport policy suggests that the climate policy consensus might be more vulnerable than has been imagined. This, plus the significant societal changes that are needed to transform UK transport in ways that are aligned with the Climate Change Act, will, it is argued, require more rather than less politics^15^. This, in turn, challenges scholars to reconsider the legitimate role academia should play in this defining issue. We see three key opportunities where there is a strong imperative and an opportunity for different or stronger engagement.

First, it is important for us, as scientists and scholars, to act as clearly independent scrutineers of the assumptions behind the watering down of climate commitments and shifts in problem framing. In so doing, we should recognise that we are active participants in the policy process and not somehow stood apart from it^16^.

Second, we need to become better at developing policy-aware and deliverable solutions and policy pathways. While much research has focused on the very substantial changes needed to remain within global carbon budgets, a huge disparity has opened up between actual policymaking today and ‘what the science tells us’ is needed. The gap between the hypothetical and the deliverable is, in part, reproduced by traditions of studying policies rather than the process and politics surrounding policy making^17,18^. Helping policy to close this gap is a critical priority if the risk of policy rejection is to be reduced.

Finally, as academic researchers, we can adapt our modes of research to become more proactive in reframing the debate, identifying solutions and challenging the downgrading of ambition^12^. As well as active engagement in public arenas, there is a range of research approaches that are oriented towards change processes and action, including transition management^19^, design experimentation^20^ and more research activist approaches^21^.

There is a spectrum of views within academia on whether taking more proactive and public positions on climate policy is a legitimate or institutionally valued role for researchers. However, the extent to which the climate policies needed to be consistent with 1.5C can survive contact with the politics of the day is becoming a defining question of the current decade. In our view, it is one with which scientists and scholars supporting such policies must engage.
